# Updates in Lung Cancer Screening: A Decade of Evidence

**DOI:** 10.1055/a-2701-9312

**Published:** 2025-10-16

**Authors:** Lori C. Sakoda, Louise M. Henderson

**Affiliations:** 1 Division of Research, Kaiser Permanente Northern California, Pleasanton, California; 2 Department of Health Systems Science, Kaiser Permanente Bernard J. Tyson School of Medicine, Pasadena, California; 3 University of North Carolina at Chapel Hill, Chapel Hill, North Carolina; 4 Lineberger Comprehensive Cancer Center, Chapel Hill, North Carolina

**Keywords:** lung cancer screening, review, randomized controlled trials

## Abstract

In this review, we summarize recent evidence from approximately the last 5 years across the lung cancer screening (LCS) care continuum. First, we review the results from the NELSON trial, from the extended follow-up of other LCS randomized controlled trials (RCTs), and from a meta-analysis of RCTs. Together, these RCTs reported a 16% relative reduction in lung cancer mortality for low-dose CT (LDCT) LCS versus non-LDCT controls. Next, we summarize updates to clinical guidelines and recommendations around LCS in the United States, noting the current debate around the use of time since quit as an eligibility criterion. We also discuss the implementation of LCS focusing on the following areas: (1) global landscape, (2) selection criteria and approach, (3) LCS program structure, (4) shared decision making, (5) smoking cessation, (6) LCS uptake, (7) American College of Radiology Lung Reporting and Data System, (8) annual LCS adherence, (9) screen-detected findings and management, (10) incidental findings and management, and (11) disparities. Lastly, we highlight emerging data and considerations for personalized LCS and new technologies, with an emphasis on risk prediction models, biomarkers, and artificial intelligence. This review highlights the latest changes to LCS and the ongoing need to monitor and evaluate LCS as it diffuses into clinical practice across various real-world settings.

Lung cancer is the leading cause of cancer incidence and mortality worldwide, with an estimated 2.4 million individuals diagnosed with and 1.8 million dying from the disease in 2022. 1 Advances in lung cancer screening (LCS) over the last two decades have the potential to alter lung cancer outcomes among populations at high risk. However, there is no universal consensus on how best to define the high-risk population that will experience a net benefit from LCS. As such, variability in clinical guidelines and recommendations for whom to screen for lung cancer exists. In addition, implementation of LCS faces numerous challenges related to the identification of eligible individuals, the use of shared decision making, and the incorporation of smoking cessation into practice. In this review, we summarize the evidence from approximately the last 5 years across the LCS care continuum, focusing on updated results from randomized controlled trials (RCTs), clinical guidelines and recommendations, implementation of LCS, and the potential for personalized LCS and new technologies.

## Lung Cancer Screening Trials

Initial results of the U.S. National Lung Screening Trial (NLST) published in 2011 reported a 20% mortality reduction in lung cancer mortality with 6.5 years of follow-up after three rounds of annual screening with low-dose CT (LDCT) compared with chest radiography among individuals ages 55 to 74 years who currently or formerly (quit within the last 15 years) smoked at least 30 pack-years (P-Y). 2 In 2020, the European NELSON trial reported a 24% reduction in lung cancer mortality with 10 years of follow-up after four rounds of LDCT among individuals ages 50 to 74 years who currently or formerly (quit within 10 years) smoked >15 cigarettes a day for >25 years or >10 cigarettes a day for >30 years. 3 The NLST and NELSON trials differed in terms of the recruited study population and nodule measurements. Compared to NLST participants, those in the NELSON trial were younger, had lower smoking intensity, and were predominantly male. In addition, NELSON assessed nodules based on volume growth, whereas NLST used nodule diameter. Despite these differences, the observed mortality reductions were similar. The NLST cohort was followed for an extended time of 11.3 years for incidence and 12.3 years for mortality outcomes, with results showing that the number needed to screen to prevent one lung cancer death was 303 and thus similar to the original analyses. 4 Beyond the NLST and the NELSON trials, 2 3 several other RCTs examining LDCT for LCS have been conducted across Europe, including the DANTE, 5 DLCST, 6 LSS, 7 LUSI, 8 MILD, 9 ITALUNG, 10 and UKLS. 11 A meta-analysis of these seven RCTs and the NLST and NELSON trials reported a 16% relative reduction in lung cancer mortality for LDCT LCS versus non-LDCT controls (relative risk [RR] = 0.84; 95% confidence interval [CI]: 076–0.92). 11

Although not powered to detect differences by subgroups, several analyses of LCS RCT results stratified by demographics suggest differences in mortality outcomes with regard to participant sex. In the NLST, the overall mortality RR was lower in women versus men (0.73 vs. 0.92, *p* -value for interaction 0.08), which appeared to be driven by differences in small cell and squamous cell carcinoma rather than nonsquamous NSCLC. 12 The NELSON trial, which included a small subsample of women, found that LDCT LCS was more favorable among women compared to men. In the German LUSI trial, women screened for lung cancer had a 69% mortality reduction (hazards ratio = 0.31; 95% CI: 0.10–0.96). 8 Pooled estimates from the NLST, NELSON, LUSI, and UKLS trial reported a 29% (RR = 0.71; 95% CI: 0.59–0.86) reduction for women and 15% for men (RR = 0.85; 95% CI: 0.76–0.95; the estimate for men also includes the DANTE trial, which excluded women). 13

## Clinical Guidelines and Recommendations

In the United States, several organizations recommend LCS based on individuals' age, smoking P-Y, years since quit (YSQ), smoking, and other factors, including comorbid conditions, functional status, or the ability to tolerate curative intent therapy ( Table 1 ). 14 15 16 17 18 19 20 The recommendations for LCS have expanded over time. For example, the U.S. Preventive Services Task Force (USPSTF) updated their 2014 recommendations in 2021 to reduce the age at which to initiate LCS from 55 to 50 years and lowered the P-Y minimum from 30 to 20. 20 21 22 23 24 25 This revision is expected to increase the eligible population by 6.4 million (81%). 21

**Table 1:** Lung cancer screening recommendations, United States

Organization	Year updated	Age, y	Pack-years	Time since quit, y	Other
American Academy of Family Physicians 14	2021	50–80	≥20	15	NA
American Association of Thoracic Surgery 15	2012	55–79	≥30	15	NA
		50–79	≥20	NA	Cumulative risk >5% over the next 5 y
		NA	NA	NA	lung cancer survivors with no incidence of disease for ≥4 y
American Cancer Society 16	2023	50–80	≥20	NA	NA
American College of Chest Physicians 17	2021	50–80	≥20	15	NA
Center for Medicare and Medicaid Services 18	2022	50–77	≥20	15	NA
National Comprehensive Cancer Network 19	2025	50+	≥20	NA	No stopping age; screening not recommended for those with functional status or comorbidity that would not allow curative intent therapy
U.S. Preventive Services Task Force 20	2021	50–80	≥20	15	NA

There is debate around the eligibility criteria for YSQ smoking. A systematic review conducted by the American Cancer Society (ACS) Cancer Related Evidence Synthesis Team found that the risk of developing lung cancer persisted beyond 15 YSQ and that risk remained significantly elevated even after quitting. 26 Based on this review and several other studies, 27 28 the ACS removed the YSQ from their LCS recommendation. 16 This change is expected to result in nearly 5 million more U.S. adults being eligible for annual LCS.

## Implementation

### Global Landscape

LCS implementation is expanding worldwide ( Table 2 ). To date, national screening programs have been established in North America (the United States), Asia (Japan, China, South Korea, and Taiwan), and Europe (Croatia, the Czech Republic, and Poland). Japan is the only country where screening has primarily occurred by chest radiology and sputum cytology until now. 29 Programs have varied in their effectiveness to reach the target population, due in part to differences in delivery, resource allocation and capacity, and policy decisions. 30 In the United States, LCS uptake remains below 20%, 31 whereas in Croatia, it has exceeded 80%. 32

**Table 2:** Global implementation of lung cancer screening

Country	Implementation stage	Key milestones	Eligible population
United States	Implemented nationally	2013: U.S. Preventive Services Task Force first recommended annual LDCT screening for high-risk adults, based on National Lung Screening Trial (NLST) results 2015: Centers for Medicare and Medicaid Services established coverage for LDCT screening	Asymptomatic adults aged 50–80 with a >20 pack-year smoking history and still smoking or quit within the last 15 y
Canada	Implemented in some, but not all provinces	2021: Ontario launched an LCDT screening program at 4 sites after an implementation pilot 2022: British Columbia launched a province-wide LDCT screening program 2024: Nova Scotia launched a province-wide LDCT screening program 2025: New Brunswick will launch a province-wide LDCT screening program Quebec, Alberta, Newfoundland, and Labrador have implemented pilot programs	Ontario: Adults aged 55–80 who have a >20 pack-year smoking history and have smoked within the last 15 y British Columbia: Adults aged 55–74 who have a >20 year smoking history and are still smoking or have quit Nova Scotia: Adults aged 50–74 who have smoked daily for 20 or more years
Japan	Implemented nationally, with opportunistic LDCT screening	1987: Japan began offering chest X-ray screening to people aged 40 and older, with opportunistic LDCT screening 2010: Ministry of Health, Labor, and Welfare initiated a nationwide randomized controlled trial comparing two LDCT scans vs. a single chest X-ray scan over a 10-y period in over 20,000 adults aged 50–70 who currently or formerly smoked. Follow-up expected to end in 2035	Adults aged 40 and older
South Korea	Implemented nationally	2015: A multi-society collective developed guidelines recommending annual LDCT screening for high-risk individuals 2017–2018: Korean Lung Cancer Screening Project (K-LUCAS) conducted to assess the feasibility of implementing a national screening program, with promising results 2019: Formal launch of national program of biannual LDCT screening, including smoking cessation counseling 2023: Over 600,000 eligible individuals screened (∼53% uptake)	Adults aged 54–74 with a smoking history of >30 pack-years
Taiwan	Implemented nationally	2014: Taiwan Lung Cancer Screening for Never-Smoker Trial (TALENT) started 2022: The Ministry of Health and Welfare launched a national program of biannual LDCT screening for high-risk populations	Two high-risk populations: 1. Adults aged 50–74 with a smoking history of >30 pack-years 2. Men aged 50–74 and women aged 45–74 with a family history of lung cancer
China	Implemented primarily in urban areas	2012: China launched the Cancer Screening Program in Urban Areas, which includes biannual LDCT screening of high-risk individuals across 75 cities in 30 provinces	Adults meeting one of the following criteria: ≥30 pack-years of smoking and <15 y since quitting; exposed to passive smoking at home or in the workplace for ≥20 y; a diagnosis of chronic obstructive pulmonary disease; >1 y of regular occupational exposure to asbestos, radon, beryllium, chromium, cadmium, nickel, silica, coal smoke, or soot; first degree family history of lung cancer
Singapore	Not implemented	No national screening program, but the Ministry of Health recommends LDCT screening for high-risk populations	
Croatia	Implemented nationally	2020: Croatia became the first European country to launch a national LDCT screening program 2023: More than 26,000 people screened, representing over 80% of the target population	Adults aged 50–75 with a smoking history of >30 pack-years and still smoking or quit in the last 15 y
The United Kingdom	Implemented nationally	2011: UK began pilot trials and studies to assess feasibility and aspects of LDCT screening, including UK Lung Cancer Screening (UKLS) pilot trial, Liverpool Health Lung Programme, Manchester Lung Health Check pilot, Lung Screen Uptake Trial (LSUT), West London lung screening pilot trial, Yorkshire Lung Screening Trial (YLST), and SUMMIT study 2019: NHS England launched the Targeted Lung Health Check program, which demonstrated efficacy to support the national LDCT screening program 2023: The UK began a national rollout of the LDCT screening program with targeted outreach, with complete rollout expected by 2030	Adults aged 55–74 who are registered with a GP practice and currently or formerly smoke are invited—eligibility for LDCT screening is then determined by model-based risk assessment
The Czech Republic	Implemented nationally via pilot	2022: The Czech Republic launched a 5-y population pilot screening program	Adults aged 55–74 with a smoking history of >20 pack-years
Poland	Implemented nationally via pilot	2018: Consensus for national LDCT screening, based on pilot data 2020–2024: Poland conducted a national pilot LDCT screening program across six macro-regions	Adults aged 55–74 with a smoking history of >20 pack-years or specific occupational exposures or related health conditions
Hungary	Assessing feasibility	2013–2020: First pilot conducted to demonstrate the feasibility of LDCT screening 2019–2022: Second pilot conducted to build a patient pathway 2023: Third pilot started to determine the approach for identifying high-risk individuals and inviting them to be screened. Data expected in 2025	
Italy	Assessing feasibility	2021: Model for Optimized Implementation of Early Lung Cancer Detection: Prospective Evaluation of Preventive Lung Health (PEOPLHE) trial launched to evaluate the feasibility and implementation of LDCT screening in the national healthcare system	
Spain	Assessing feasibility	2022: Spain launched the CASSANDRA Project to explore the feasibility of implementing LDCT screening	
France	Assessing feasibility	2022: The French Health Authority acknowledged the efficacy of LDCT screening and recommended a national pilot program 2025: Launch of pilot program to assess the feasibility of national LDCT screening implementation	
Germany	Assessing feasibility	2024: Federal Ministry for the Environment, Nature Conservation, Nuclear Safety, and Consumer Protection issued the Lung Cancer Early Detection Ordinance, allowing providers to offer LDCT screening	
Belgium	Assessing feasibility	2025: Flanders will launch an implementation study on LDCT screening in Zuid-Oost Rand Antwerpen (ZORALCS)	
The Netherlands	Assessing feasibility	Research and implementation trials, including the 4-IN-THE-LUNG-RUN (4ITLR) trial, are ongoing to examine aspects of LDCT screening implementation	
Australia	Pending implementation	2025: Australia will launch a national program of biannual LDCT screening in July	Asymptomatic adults aged 50–70 with >30 pack-year smoking history and still smoking or quit within the last 10 y

Other countries are at varying stages of implementation. In Canada, several provinces have implemented LCS programs, while other provinces are running pilot programs. 30 Implementation in Europe has occurred slowly, despite strong evidence from NELSON and other European screening trials and implementation studies. In September 2022, the European Commission endorsed stepwise implementation of LCS across the European Union (EU), and shortly thereafter, the EU Council of Ministers for Health approved funding for LCS at the national and EU levels. The Strengthening the Screening of Lung Cancer in Europe (SOLACE) project, which launched in April 2023, has become a key initiative within Europe's Beating Cancer Plan. SOLACE is uniting a network of European respiratory and radiology experts from 15 countries to enhance LCS efforts in underserved and high-risk populations across the EU. 33 Also in September 2022, the United Kingdom National Screening Committee recommended LCS, with a policy review outlining key priorities and requirements for implementation. 34 The UK began rolling out its nationwide targeted LCS program in 2023, with the goal of reaching 40% of the eligible population by 2025 and full implementation by 2030. 35 Australia is launching its national screening program in July 2025. 36

Initiatives dedicated to advancing high-quality LCS implementation include the Lung Cancer Policy Network, 32 the G7 Cancer Alliance, 37 and the ACS National Lung Cancer Roundtable (NLCRT). 38

### Selection Criteria and Approach

No universal consensus exists on defining the population at “high risk” who is most likely to benefit from LCS. Accordingly, the population eligible for LCS differs between countries ( Table 2 ). Even within the screening-eligible population, individual risk for developing lung cancer varies substantially, and the benefit conferred by LCS varies according to risk. 22 The selection criteria largely center on age and smoking history, given that lung cancer risk strongly increases with greater age and tobacco exposure, with some variation in the age range and thresholds for P-Y smoked and time since quit applied across countries. Selecting individuals on fixed age and smoking history criteria alone; however, results in including some at low risk who are unlikely to benefit from LCS and excluding others at high risk who are likely to benefit from LCS. 39 40 Some countries consider screening based on other criteria. In Japan, eligibility is based on age alone. 29 Taiwan is the first to include screening of individuals with a family history of lung cancer who have never smoked, 41 while China is the first to include screening of individuals with a history of chronic obstructive pulmonary disease (COPD), with specific occupational exposures, or with a family history of lung cancer. 42 Poland has also included screening individuals with specific occupational exposures or related health conditions. 43

Risk-based screening is an alternative approach with the potential to improve the balance of benefits to harms by screening fewer individuals, producing fewer false-positive results, and detecting more early-stage lung tumors. 44 Individuals are selected based on whether their personal risk for lung cancer—calculated using risk prediction models that incorporate age, smoking history, and various clinical and nonclinical factors—exceeds a specified risk threshold. To date, a myriad of risk prediction models have been proposed to estimate individual risk of developing or dying from lung cancer within a given time window; yet, only a handful have exhibited good performance in external validation studies 45 : the Bach model, 46 PLCO _M2012_ model, 47 Liverpool Lung Project (LLP) model, 48 49 Lung Cancer Risk Assessment Tool, 50 and Lung Cancer Death Risk Assessment Tool. 50

Retrospective analyses consistently show that applying accurate risk prediction models to select ever-smoking adults for screening is more effective in preventing lung cancer deaths than applying fixed criteria on age and smoking history. 23 47 50 51 Studies also suggest that risk-based screening using the PLCO _M2012_ model can reduce lung cancer disparities, with a higher sensitivity for detecting lung cancer in racial and ethnic minority populations and women than USPSTF criteria-based screening. 52 53 54 However, risk-based screening can lead to comparatively modest increases in the number of life years and quality-adjusted life years (QALYs) gained and greater overdiagnosis, because it preferentially selects adults at the highest risk; these adults are generally older, have more comorbidities, and thereby may benefit less due to their shorter life expectancy and higher mortality from other causes. 51 55 56 Ideally, selecting individuals for LCS would consider both individual estimates of risk and life expectancy to prevent the most deaths and maximize the expected life-years gained in the population. 39 55 57 Several novel frameworks and strategies that incorporate individual risk, preferences, and life expectancy have been developed to inform risk-based screening decisions, although the minimum gain in life expectancy to recommend remains unclear. 55 58 59 Also under specific modeling assumptions, risk-based screening strategies appear more cost-effective than screening based on the 2021 USPSTF criteria. 60

Outside the United States, promising results have emerged from multiple prospective trials and studies evaluating the feasibility and effectiveness of implementing risk-based LCS. The SUMMIT study demonstrated the feasibility of effectively delivering large-scale LCS to 12,773 enrolled the UK participants aged 55 to 77 who either met the 2013 USPSTF criteria or had a PLCO _M2012_ risk threshold of ≥1.3%. 61 62 The Manchester Lung Health Check pilot was likewise successful in delivering targeted LCS to high-risk individuals with a PLCO _M2012_ risk threshold of ≥1.51% living in sociodemographically disadvantaged areas. 63 64 In this pilot, follow-up of the screened and unscreened groups, classified based on the PLCO _M2012_ risk threshold, confirmed that only a few lung cancer cases arose in the low-risk, unscreened group. 65 The Yorkshire Lung Screening Trial (YLST) compared the performance of selecting high-risk participants with the PLCO _M2012_ risk threshold of ≥1.51%, LLP _V2_ risk threshold of ≥5%, and 2013 USPSTF criteria. 66 The PLCO _M2012_ model identified the most people eligible for screening and the most screen-detected lung cancers, and both risk models were more efficient at selecting individuals than the USPSTF criteria. 67 While telephone risk assessment was effective in inviting individuals for LCS in the YLST, current smoking status and socioeconomic deprivation corresponded with lower participation. 68 Interim analyses of the International Lung Screening Trial (ILST) further suggest that selecting ever-smoking adults aged 55 to 80 using the PLCO _M2012_ risk threshold of ≥1.51% is more effective than the 2013 USPSTF criteria. 69 70 Cost-effectiveness analyses using ILST data also show that risk-based screening results in greater cost savings, increased QALYs, and reduced inequities in screening access. 71 Currently, the 4-IN-THE-RUN trial is recruiting 900,000 individuals with a smoking history of ≥35 P-Y who have smoked in the last 10 years or with a PLCO _M2012_ risk of ≥2.6% from the Netherlands, Germany, England, France, Italy, and Spain to determine the optimal personalized LCS approach that incorporates comorbidity-reducing strategies. 72

### Program Structure

In the United States, LCS programs are generally structured using a centralized, decentralized, or hybrid approach. 17 LCS guidelines do not recommend a specific approach, but instead the approach that aligns best with existing resources and needs of the population served. In centralized programs, primary care providers and other clinicians refer individuals to an LCS program, which has dedicated personnel responsible for critical program components, including eligibility assessment, shared decision-making (SDM), tobacco cessation management, LDCT ordering, communication of LDCT results, and management of follow-up screening and care. In decentralized programs, ordering providers hold overall responsibility for the entire LCS process, including specialty referral for follow-up care. At present, the majority of LCS occurs in decentralized settings. Many programs are also hybrid, where some but not all elements are centralized along the LCS continuum.

Centralized programs appear to be more effective, particularly in terms of adherence to annual LCS and follow-up care, than decentralized programs. 73 74 75 Centralized programs also more commonly implement practices to support LCS before screening is initiated. 76 Yet, the specific structures and practices of centralized programs that enhance LCS quality and effectiveness remain unclear. 77 78 Limited evidence suggests that patient navigation practices, including support with scheduling, transportation, and accessing resources to reduce barriers to care, can improve LCS uptake and adherence, particularly in vulnerable populations. 79 80 81 Given that centralized programs require considerably more investment and resources, further research is needed to understand which components of centralized programs are the most beneficial and whether they could be extended into noncentralized programs. 77 78

### Shared Decision-Making

SDM is recommended to help patients in making informed decisions about LCS with clinicians, considering the best available evidence on the benefits and harms of LCS and their own values and preferences. 20 The American Thoracic Society and Veterans Affairs Health Services Research and Development jointly advocate that SDM embrace several core principles, specifically to “empower the patient to participate in SDM to the extent they desire,” “include the information about LCS that the patient needs and wants to make an informed, value-based decision,” and “avoid exacerbating population-level disparities or worsening stigma related to smoking.” 82 For reimbursement coverage, the U.S. Centers for Medicare and Medicaid Services mandates a documented SDM visit with a qualified healthcare provider (physician, physician assistant, or nurse practitioner) before the beneficiary's first LDCT scan. 18 Specific visit requirements entail eligibility determination, SDM that incorporates the use of at least one decision aid, as well as counseling on the importance of screening adherence and of smoking cessation and abstinence, the impact of comorbidities, and the willingness to undergo follow-up care and treatment if abnormal findings arise.

These standards have been challenging to meet in practice. Although clinicians recognize the value of SDM, many cite major barriers to facilitating SDM, including time constraints, competing clinical demands and priorities, limited awareness about LCS guidelines, lack of skilled training in SDM, and insufficient resources to implement SDM. 83 84 85 Concerns have been raised about the intent and quality of SDM for LCS, given evidence that SDM conversations are often brief, prioritize exchanging information more than eliciting patient preferences, focus on benefits over harms, and lack the use of decision aids. 85 86 Nonetheless, various decision aids, tools, and processes for SDM have been reported to improve patient knowledge, reduce decisional conflict, show acceptability to patients and providers, and increase LCS uptake and adherence. 87 88 89 90 91 92 Especially those effectively tailored to populations at higher risk for lung cancer, such as people with HIV, may further promote health equity. 92 93

The role of SDM has become even more important since the USPSTF expanded the screening-eligible population to include individuals at lower absolute risk of lung cancer. Yet, the optimal approach to achieving high-quality SDM in LCS remains elusive. To overcome clinician barriers, a practical approach would have well-trained nonclinicians conduct SDM. 94 Several effectiveness-implementation studies are ongoing to identify effective and scalable approaches for SDM in LCS, including the TELESCOPE study that is evaluating a telehealth decision coaching and navigation intervention delivered by patient navigators in primary care clinics. 95 96 97 Clinicians are also open to employing more novel approaches, such as prediction-augmented SDM tools, which provide tailored LCS recommendations according to the level of predicted benefit. 98 Priorities outlined by the NLCRT and others to advance SDM research and implementation in LCS include developing adaptable SDM training programs for health care personnel, understanding how alternative screening delivery models affect SDM quality, developing and evaluating novel SDM tools across different settings and populations, establishing key SDM process quality measures, and investigating the utility of prediction-driven SDM. 82 99

### Smoking Cessation

Smoking cessation is a necessary, but challenging component of high-quality LCS. 100 With at least 50% of all screen-eligible individuals actively smoking, 101 LCS offers “teachable moments” to reinforce smoking abstinence and to motivate and support those who smoke to quit. 102 103 Of the participants in the UK Lung Health Check program who currently smoked, 44% indicated that screening made them consider quitting, 29% indicated that it made them attempt to quit, and 25% indicated that it made them smoke less, although only 10% indicated that it made them seek help to quit. 104

Among NLST participants from the American College of Radiology (ACR) Imaging Network arm, over a third were highly addicted to nicotine and reported smoking within 5 minutes of waking up. 105 Yet, of those participants who smoked at enrollment and underwent LCS, 73.4% received no pharmacologic tobacco treatment, and those who were female, African American, unmarried, and less educated were less likely to attempt quitting. 106 Other studies have similarly noted that lower nicotine dependence and lower educational attainment correspond to less engagement in smoking cessation interventions. 107 108 109

Model-based analyses indicate that smoking cessation confers added population health benefit beyond LCS alone. Even with modest quit rates (e.g., 10% quit), a single smoking cessation intervention at the initial screen can lead to fewer lung cancer deaths and notable gains in life expectancy, since smoking cessation reduces the risk of developing lung cancer and other tobacco-related conditions. 110 Additionally, integrating smoking cessation interventions, such as telephone-based counseling with nicotine replacement therapy (NRT), for individuals engaged in LCS has been estimated to be more cost-effective than LCS alone. 111 112 113

As aforementioned, counseling on smoking cessation and interventions is recommended as part of the SDM visit. Beyond this, no specific guidance is provided on how to optimally deliver cessation interventions within the LCS process. The Society for Research on Nicotine and Tobacco and the Association for the Treatment of Tobacco Use and Dependence mutually recommend that individuals who smoke should be encouraged to quit at every screening visit and be offered evidence-based smoking cessation interventions, irrespective of their scan results or motivation to quit. These interventions should also be offered by SDM or other qualified providers; otherwise, individuals should be directed to tobacco cessation services. 114 However, many barriers hinder effective implementation of these services, including a lack of organizational support and limited time and reimbursement allotted to providers for treatment. 115 Most SDM providers further lack specialized training needed to provide comprehensive smoking cessation support. 116 Given these constraints, the most effective cessation intervention to implement in any given LCS program is likely to depend on its population, setting, and institutional resources and screening workflows. 117

There is limited but growing evidence on best practices to optimize the delivery of smoking cessation interventions in LCS programs. In 2016, the U.S. National Cancer Institute established the Smoking Cessation at Lung Examination (SCALE) Collaboration, an initiative comprised of eight clinical trials evaluating the efficacy of smoking cessation interventions in the context of LCS. These trials tested various approaches ranging from individual therapies to health system interventions, and they collectively recruited 5,752 participants at 76 clinics. 118 Compared to screen-eligible individuals who smoke in the general U.S. population, participants enrolled in seven SCALE trials smoked slightly less, but were demographically similar at baseline. 119 Results from six SCALE trials have been published ( Table 3 ). 120 121 122 123 124 125 126

**Table 3:** Primary findings of U.S. scale trials

Trial	Primary findings
Georgetown Lung Screening, Tobacco, and Health (LSTH) trial 120	In this randomized trial of 818 participants, those who received the intensive (8 telephone counseling sessions with 8 wk of nicotine patch) vs. minimal (3 telephone counseling sessions with 2 wk of nicotine patch) intervention had a higher self-reported quit rate at 3 mo (14.3 vs. 7.9%), but not at 6 or 12 mo
Optimizing Lung Screening Intervention (OaSIS) trial 121	This effectiveness-implementation cluster randomized trial of 26 radiology facilities across 20 U.S. states found that average tobacco cessation increased from 0% at baseline to 13% at 6 mo but did not differ by trial group (intervention vs. usual care) at any measured timepoint (14 d, 3 mo, and 6 mo)
Program for Lung Cancer Screening and Tobacco Cessation (PLUTO) trial 122	This adaptive sequential, multiple assignment randomized trial enrolling 636 screen-eligible adults who smoked daily from three large health systems found that adding a prescription medication therapy management referral to a tobacco longitudinal care (TLC) program (involving intensive telephone coaching and combination NRT for 1 y with at least monthly contact) did not improve smoking abstinence at 18 mo among those who did not respond to early treatment. In addition, the deintensification of TLC to quarterly contact among those who responded to early treatment had an unfavorable effect on smoking abstinence
Personalized Intervention Program: Tobacco Treatment for Patients at Risk for Lung Cancer (PIP) trial 123	This two-phase, sequential, randomized controlled trial enrolling 188 patients found no difference in smoking abstinence at 8 wk, comparing standard of care (5 in-person counseling sessions and 8 wk of nicotine patch) plus gain-framed messaging vs. standard of care alone. Additionally, participants randomized to receive vs. not receive feedback on smoking-related biomarkers reported a similar daily number of cigarettes smoked at 6 mo
Screen ASSIST (aiding screening support in stopping tobacco) trial 124 125	In this randomized 2 × 2 × 2 factorial trial, 642 English- or Spanish-speaking adults scheduled for lung cancer screening were assigned to 8 groups receiving a multicomponent intervention with 3 treatment factors: duration of telehealth counseling (4 sessions over 4 wk vs. 8 sessions over 12 wk), duration of free NRT (2 wk vs. 8 wk), and screening for social determinants of health and referral to community-based resources (yes vs. no). At 6 mo, 7-d smoking abstinence was higher for those who received a longer duration of counseling only; no differences were noted for the other factors. This trial also found that proactive outreach at multiple points in the screening process is beneficial for engaging individuals in receiving tobacco treatment
Project LUNA 126	This trial randomized 630 screening-eligible adults who currently smoke into three treatment groups: quitline referral for counseling and 12-wk NRT; quitline referral for counseling plus 12-wk NRT or pharmacotherapy prescribed by the screening clinician; and integrated care of 12-wk NRT or pharmacotherapy and intensive counseling provided by dedicated tobacco treatment specialists. Participants who received integrated care had the highest 7-d smoking abstinence at 3 mo (37.1%), followed by those who received quitline counseling with medication (27.1%) or without medication (25.2%); however, the difference in abstinence between those who received integrated care vs. quitline counseling with medication decreased at 6 mo (32.4 vs. 27.6%)

While not all SCALE trials identified group differences for their primary outcomes, they demonstrated the feasibility of integrating various smoking cessation interventions into LCS in different settings, as well as the importance of providing longitudinal behavioral and medication support for cessation. In the UK QuILT trial that randomized 412 adults aged 55 to 75 attending a targeted lung health check, those who received immediate support from a trained smoking cessation counselor with pharmacotherapy versus usual care (brief advice for quitting and signposting to cessation services) had higher 3-month quit rates (29.2 vs. 11%). 127 A meta-analysis of 10 RCTs of smoking cessation interventions delivered with LCS (including LSTH and QuILT) suggests that more intensive interventions are the most effective relative to usual care. 128 Unequivocally, continued efforts are vital to reducing barriers to smoking cessation and implementing evidence-based strategies that support individuals to quit smoking.

### Lung Cancer Screening Uptake

Despite strong evidence that annual LCS with LDCT reduces mortality and recommendations endorsing LCS from multiple organizations, LCS uptake remains low in the United States, with 16.4% (95% CI: 15.6–17.2) of the 13.5 million eligible individuals screened in 2022, 31 the last year for which national data are available. Across the United States, LCS rates have ranged from 8.6 to 28.7%, with Northeastern and mid-Atlantic states tending to have higher rates. Currently, national self-reported LCS rates are available from the Centers for Disease Control and Prevention's Behavioral Risk Factor Surveillance System (BRFSS) through an LCS module that was optional until 2022. In 2022, the BRFSS LCS question asked if the respondent had any CT scans of the chest to check or screen for lung cancer. 129 No studies have validated the BRFSS LCS survey question, and it is possible that responders answer yes to include both screening and diagnostic chest CTs or that they may not recall accurately, both of which would result in overestimation of LCS uptake. The National Health Interview Survey 130 and the Health Information Trends Survey 131 also have cancer control supplemental modules and, in some years, asked about LCS.

While administrative claims data are also used to derive estimates of LCS uptake, these data do not contain sufficient detail to ascertain P-Y or YSQ and are limited to individuals with health insurance. 132 Electronic health records have also been used to assess LCS uptake and tend to have more, but not necessarily sufficient details on smoking information, such as P-Y and YSQ.

Several factors are associated with increased LCS uptake. A meta-analysis examining predictors of LCS in the United States found that Black or Hispanic adults had lower rates of LCS than White adults. 133 It also found geographic differences with higher rates in the Northeast and differences by socioeconomic status. The presence of comorbid conditions, particularly COPD, was also associated with higher rates of LCS uptake.

Interventions to increase LCS uptake have focused on (1) improving the identification of eligible individuals; (2) education to the community and providers; and (3) navigation to services. 134 A recent review noted that most LCS interventions address SDM and initial LCS uptake rather than LCS eligibility assessment, annual adherence, or diagnostic follow-up of screen-detected findings. 135

### Lung CT Screening Reporting and Data System

To facilitate standardized reporting and management of findings from LDCT screening exams, the ACR first released the Lung CT Screening Reporting and Data System (Lung-RADS) in 2014 (v1.0), with an update to v1.1 in 2019 and an update to Lung-RADS v2022 in November 2022. 136 The most recent updates include new classification criteria of atypical pulmonary cysts, juxtapleural nodules, airway-centered nodules, and inflammatory or infectious findings. Additionally, the latest Lung-RADS version provides clarification around volumetrics, nodule growth, the “S” modifier, and stepped management of nodules that are stable or decreasing in size. Lung-RADS continues to evolve over time based on new data and clinical insights.

### Annual Adherence

For LCS to be effective in reducing mortality, adherence to recommended screening intervals is needed. Among individuals with a negative or normal LCS exam, the Lung-RADS recommendation is to return for screening annually. Measuring annual adherence across studies is challenging due to differences in definitions around the timing of follow-up and a need for standardization. 137

Numerous studies have evaluated annual adherence to LCS. 73 74 138 139 140 141 142 143 144 145 A meta-analysis by Lopez-Olivo et al reported a pooled LCS adherence rate of 55% (95% CI: 44–66), with adherence rates across studies ranging from 12 to 91%. 143 The wide variability in rates is likely due to differences in definitions, study populations, and study settings. Results of the pooled analyses showed factors associated with higher rates of annual adherence include centralized versus decentralized screening programs, having formerly smoked versus currently smoking, White versus other races, and completing 4 years or more of college versus not. A recent multicenter study of 10,170 individuals screened for lung cancer found that adherence to annual LCS was associated with increased lung cancer detection, especially at earlier stages; however, adherence declined annually following baseline screening, further signifying the need to improve adherence. 140

### Screen-Detected Findings and Management

Effective follow-up and management of pulmonary nodules detected from LCS is paramount to realizing the reported net benefits of LCS observed in the RCTs. Lung-RADS recommends specific testing and time intervals for follow-up depending on pulmonary nodule size, attenuation, shape, margin, calcification, and growth rate. Retrospective application of Lung-RADS criteria to the NLST resulted in 13.7% of baseline exams and 5.8% of subsequent exams classified as positive and warranting additional follow-up. 146 In real-world settings, approximately 11.4 to 19.6% of LCS exams are classified as positive (Lung-RADS 3, 4A, 4B, or 4X). 147 148 149 150 151 152 In a multi-center cohort study, absolute rates of downstream imaging and invasive procedures were 31.9 and 2.8%, respectively. 152

Limited data indicate suboptimal adherence to recommended follow-up care following positive LCS findings. In a prospective U.S. cohort study, overall adherence to recommended follow-up care was 42.6%, with an increasing trend in adherence from Lung-RADS category 3 to 4B/4X (30–68%), and factors related to poorer adherence included Black race, male sex, and current smoking status. 153 Another study similarly noted that almost half of the patients had delayed follow-up care after positive findings (i.e., >30 days beyond recommended Lung-RADS intervals), and among those diagnosed with lung cancer, delayed follow-up care was associated with clinical upstaging. 154

Studies have documented higher rates of malignancy with increasing Lung-RADS category. In one study, malignancy rates corresponding to Lung-RADS categories 3, 4A, 4B, and 4X were 3.9, 15.5, 36.6, and 76.8%, respectively. 155 Lung cancer detection rates by LDCT LCS have ranged from 0.56 to 4.5%. 156 157 158 159

### Incidental Findings and Management

Incidental findings (IFs) detected on LDCT LCS are defined as imaging abnormalities unrelated to lung cancer and are both pulmonary and extrapulmonary in nature. While some IFs may be benign, other IFs may be clinically significant (e.g., significant incidental findings [SIF]) and require subsequent follow-up, including diagnostic work-up and management. In 2023, the ACR released a quick reference guide for IF detected on LCS, which gives an overview of the most common IFs. 160

Among the LDCT screening arm of the NLST, 18% of exams had at least one SIF, with the most common types being pulmonary findings (43%), coronary artery calcification (CAC; 12%), and masses or suspicious lesions (7%). 161 SIF were reported for about 8% of participants in the NELSON and COSMOS trials and for about 10% of those in the Australian and Canadian ILST. 162 163 164 Similar to the NLST, the most frequent IFs were CAC and emphysema.

There is limited research on IF detected on LCS outside of RCTs. 165 In real-world U.S. settings, Lung-RADS specifies using an “S” modifier to indicate a clinically or potentially clinically significant IF that is unrelated to pulmonary nodules. However, prior studies have shown that the “S” modifier does not capture all IF for which clinical follow-up is recommended. Hence, studies estimating SIF prevalence have applied differing definitions and have reported wide variation ranging from 16 to 69%. 166 167 168 169 170 171 A scoping review of 32 articles examining CAC detected from LCS found CAC prevalence of 14.8 to 98%. 172

The net benefits of detecting SIF on LDCT LCS are unknown. While detecting clinically actionable findings has been shown to favorably impact outcomes, there is also the potential to detect nonsignificant IF that may cause unneeded anxiety, unnecessary imaging or invasive procedures, or financial distress. This poses challenges for patients, providers, systems, and payors. Additional research to evaluate the clinical benefits and harms of SIF detected on LCS is needed.

### Disparities

Established drivers of LCS disparities include healthcare access and costs. 173 Accessibility to LCS intrinsically depends on travel distance to LDCT facilities, with distance being inversely associated with population density and urbanization. 174 175 Geospatial analyses estimate that 5% of the eligible U.S. population has no access to any LDCT facility within 40 miles, and that lack of access is especially pronounced in rural and socioeconomically disadvantaged areas and populations. 175 176 177 178 179 180

LCS utilization is further influenced by health insurance access and coverage. Analyses using the BRFSS survey data have found a greater proportion of screened non-Hispanic Black respondents were of Medicare age, despite similar LCS rates between non-Hispanic White and Black respondents, 181 and that gaining access to Medicare coverage increased LCS in screening-eligible men. 182 Additionally, U.S. claims data suggest that individuals with higher total out-of-pocket medical costs are less likely to adhere to annual LCS. 183

To date, the few interventions addressing social determinants of health in LCS have favorably resulted in increased screening rates, as shown for other screening-associated cancers. 184 Various proposed opportunities to mitigate disparities include increasing public awareness about LCS, addressing misconceptions and stigma associated with lung cancer, establishing mobile LCS clinics in underserved communities, and fostering partnerships for community engagement. 185 186

## Personalized Screening and New Technologies: Emerging Data and Considerations

Applying personalized approaches holds great promise for enhancing the effectiveness, efficiency, and accessibility of LCS. A growing body of research focuses on integrating risk prediction modeling, biomarkers, and artificial intelligence (AI) into LCS. Although emerging data are encouraging, including that primary care clinicians are open to adopting personalized LCS, 187 most proposed innovations require more robust evaluation and consideration before widespread clinical implementation.

### Risk Prediction Models

The application of lung cancer risk prediction models has been largely focused on tailoring the selection of high-risk adults who have smoked for LCS. A major consideration is the optimal risk threshold upon which to screen. This choice should strike the best balance between individual benefits and harms along with program efficiency, costs, and impact. 188 Although adopted, the PLCO _M2012_ 6-year risk threshold of ≥1.51% was not derived considering cost-effectiveness. 189 Optimal risk thresholds must be set for each model, as different models generate varying absolute risk estimates for the same individual. 190 Risk thresholds must also be reassessed and adjusted as population characteristics change over time. 57

Evidence increasingly supports using the PLCO _M2012_ and LLP _V2_ models in targeted LCS programs. 191 However, the clinical feasibility and acceptability of newer models, especially ones designed to incorporate comorbidities, life expectancy, and/or individual preferences, remain largely untested. 59 Accurate collection of required input data is critical for effective implementation of model-based risk assessment. Given the time constraints clinicians face, embedding automated risk calculation within electronic health records may help to streamline workflows and promote wider adoption of personalized LCS. Additionally, developing best practices to prevent unnecessary screening of low-risk populations is needed.

Validated risk prediction models can perform differently when applied to different populations and settings. Models that perform well in predicting lung cancer risk of ever-smoking adults in Western populations have been found to perform suboptimally when applied to ever-smoking adults in Asian populations. 192 Also in Asia, approximately 30 to 40% of all lung cancers arise in adults who have never smoked, a higher proportion than observed in the United States and Europe, highlighting the need to consider factors beyond age and smoking history in assessing risk. 193 While extending models to identify high-risk adults who have never smoked may be beneficial in broadening the reach of LCS, more definitive evidence is required to support tailored screening of never-smoking adults across diverse populations.

### Biomarkers

Biomarkers can facilitate early detection of lung cancer by indicating the presence of associated molecular alterations before clinical symptoms manifest. Various lung cancer biomarkers are under investigation for their use with LDCT to refine the selection of individuals for LCS or improve risk stratification after lung nodule detection, as well as their standalone use to screen for lung and other cancers concurrently.

Blood-based biomarkers are the furthest along in clinical development, although relatively few have moved beyond early evaluation.

### Autoantibodies

In early studies, the EarlyCDT-Lung test, an assay profiling seven tumor-associated autoantibodies, 194 demonstrated high specificity (91%) and low sensitivity (34–37%), with better performance for early-stage lung cancer, and its ability to differentiate malignant from benign pulmonary nodules. 195 196 197 198 The Early Diagnosis of Lung Cancer Scotland trial randomized over 12,000 high-risk participants to receive the EarlyCDT-Lung test or usual care, and test-positive participants received LDCT every 6 months up to 2 years. More early-stage lung cancers were diagnosed in the intervention than the control arm, with no difference in mortality after 2 years 199 ; however, 5-year mortality was lower in those tested (vs. untested) for autoantibodies and diagnosed with lung cancer within 2 years. 200 As designed, the trial could not assess the added value of the EarlyCDT-Lung test to LDCT, only its benefit to select individuals for LDCT screening.

### Proteins

Nodify XL2 is a plasma-based test to identify likely benign lung nodules, integrating measurement of two proteins (LG3BP and C163A) with five clinical factors (age, smoking status, nodule diameter, nodule edge characteristics, and nodule location). It was validated in the PANOPTIC trial for 8 to 30 mm lung nodules with a pretest probability of cancer of ≤50%, achieving 97% sensitivity, 44% specificity, and 98% negative predictive value. 201 Its clinical utility in managing new solid lung nodules of low to moderate cancer risk is presently under evaluation in a multisite randomized trial (NCT04171492). This test is commercially available and covered by Medicare for certain patients with lung nodules. Additionally, a four-marker protein panel (4MP; CEA, Cyfra21-1, CA125, and Pro-SFTBP) combined with smoking history has shown greater sensitivity (63%) compared to smoking history alone (43%) in selecting individuals for LCS. 202 The 4MP in combination with the PLCO _M2012_ model has further demonstrated improved risk assessment for LCS, relative to the 2021 USPSTF LCS eligibility criteria. 203

### MicroRNAs

A plasma-based 24-miRNA signature classifier and a serum-based 13-miRNA signature test have demonstrated the potential to reduce LDCT false-positive rates with high sensitivity (78–87%) and specificity (75–81%). 204 205 206 In the BioMILD trial of over 4,000 heavy-smoking individuals, the 24-miRNA signature classifier was used conjunctively with baseline LDCT to personalize LCS intervals, showing that individuals classified as positive on both LDCT and miRNA signature had a higher 4-year lung cancer incidence and 5-year lung cancer mortality. 206 The COSMOS II study is prospectively examining the 13-miRNA signature test alongside LDCT in approximately 10,000 high-risk individuals. 207 Other miRNA panels have been examined in selecting individuals for LCS 208 and assessing the risk of solitary pulmonary nodules. 209

### Circulating DNA

Cell-free DNA (cfDNA) analysis is a major focus of investigation in multicancer and LCS. The commercially available Galleri test, which analyzes cfDNA methylation patterns, initially exhibited 55% sensitivity and 99% specificity for detecting >50 cancer types across all stages, with about 20% sensitivity for early-stage lung cancer. 210 This test is currently being validated for detecting multiple cancers at an early stage in over 13,000 UK SUMMIT participants at high-risk for lung cancer (NCT03934866). Its safety and performance are also under evaluation in PATHFINDER 2, a prospective interventional study comprising 35,000 U.S. adults eligible for cancer screening (NCT05155605). Based on analyses from the Copenhagen City Heart Study, DNA methylation could also be used with existing screening criteria to enhance the selection of individuals for LCS by excluding those at the lowest risk. 211 DELPHI (DNA evaluation of fragments for early interception) analyzes genomewide cfDNA fragmentation profiles to detect cancer. 212 The CASCADE-LUNG study is underway to prospectively validate DELPHI test performance in detecting lung cancer among nearly 12,000 screening-eligible adults (NCT04825834). The FIRSTLUNG cluster randomized trial is further assessing the clinical utility of DELFI on promoting LCS uptake in primary care settings (NCT0614570).

Beyond blood, other promising sources of biomarkers include airway epithelia, exhaled breath, sputum, and urine. Yet, some constraints hinder the utility and application of biomarkers in LCS. 213 214 Blood and sputum tests have been generally limited by low sensitivity, especially for detecting small tumors. Assessing airway epithelia via bronchoscopic brushing is invasive. Methods for sampling and analyzing volatile organic compounds in exhaled breath lack standardization. Urine biomarkers can be influenced by diet, medication, and comorbidities. Most notably, many biomarkers have yet to undergo rigorous prospective validation for their intended use in LCS.

### AI

With LCS implementation, radiologists face greater demands to interpret higher volumes of CT images promptly and accurately. AI models developed for pulmonary nodule detection and classification show promise in overcoming this challenge through automating image analysis and reducing inter-reader variation. Compared to radiologists, AI models for nodule detection have exhibited higher sensitivity (86–98 vs. 68–76%) but lower specificity (78–87 vs. 87–92%), and those for classification of nodule malignancy have generally exhibited better sensitivity (61–93 vs. 77–88%), specificity (64–96 vs. 62–84%), and accuracy (65–92 vs. 73–86%). 215 Evaluating LDCT interpretation times and outcomes across simulated AI use case scenarios, using AI as a prescreener (i.e., radiologists only interpret exams with a positive AI result) was the only scenario that led to a lower recall rate (20.8 vs. 22.1%), lower mean interpretation time (143 seconds vs. 164 seconds), and higher per-exam specificity (90.3 vs. 88.8%), relative to radiologist interpretation without AI. 216 Other scenarios included using AI as an assistant (i.e., radiologists interpret all exams with AI assistance) and using AI as a backup (i.e., radiologists reinterpret exams when AI indicates a missed finding). Data from the 4-IN-THE-LUNG-RUN and UK LCS trials further suggest that using commercial AI software as a first read to independently rule out negative LDCT scans at baseline can outperform radiologists and reduce their workload, without considerably missing diagnostic referrals or lung cancers. 217 218

AI models that predict future lung cancer risk have primarily emerged since 2020, exhibiting better performance than traditional regression-based models. 219 While most traditional risk prediction models consider epidemiologic and clinical factors, several AI models consider imaging data only. Deep learning models incorporating imaging data from LDCT scans, such as the Ardila et al 220 and Sybil 221 models, have especially shown strong predictive performance (pooled area under the curve: 0.85; 95% CI: 0.82–0.88), offering the potential to tailor screening frequency to an individual's predicted lung cancer risk from baseline or subsequent LDCT scans. 219 Notably, Sybil predicts risk from a single LDCT scan without additional clinical data or radiologist annotations, has been externally validated in several populations, and is publicly accessible. 221 AI can further support opportunistic CT screening for other chronic conditions, including cardiovascular disease, emphysema, and osteopenia, as an added benefit toward improving population health. 222

To advance integration of AI-based tools in LCS, large-scale prospective validation of their performance and clinical utility is especially needed in diverse populations. That encompasses examining how imaging parameters and reconstruction techniques influence their performance, given variability in the imaging scanners and protocols used across clinical settings, as well as evaluating how AI-based tools impact the performance of radiologists. Investigating the interpretability of results from deep learning models through correlation with input data is also important to better understand how predictions are made. 223 Although relatively in its infancy, AI shows immense potential to transform LCS, particularly in facilitating clinical decision-making.

## Conclusion

Over the last 5 years, the landscape around LCS has changed. Updated data from LCS RCTs solidify the mortality benefit of LDCT for LCS among high-risk individuals, especially with data from the NELSON study supporting LCS among younger individuals with lower smoking intensity. In the United States, updated LCS guidelines have reduced the age at which to initiate LCS to 50 years and the minimum smoking intensity to 20 P-Y, with some differences in guidelines around the time since quit criteria and the incorporation of other risk factors. While retrospective analyses consistently show that selecting ever-smoking adults for LCS based on risk prediction models is more effective than applying fixed criteria based on age and smoking history, implementation of risk prediction models into practice is challenging but possible. The SCALE and other trials demonstrated the feasibility of integrating various smoking cessation interventions into LCS across different settings and reinforced the importance of providing longitudinal behavioral and medication support for smoking cessation. In real-world settings, LCS uptake and adherence remain suboptimal, and lung cancer detection rates with LDCT LCS range from 0.56 to 4.5%. There is limited data on the net benefits of IFs detected on LCS. While the use of personalized approaches to LCS holds great promise for enhancing the effectiveness, efficiency, and accessibility of LCS, most proposed innovations require additional evaluation and consideration before widespread clinical adoption.
